# Effects of hydromorphone-based patient-controlled intravenous analgesia on postpartum depression after cesarean section: a prospective randomized controlled trial

**DOI:** 10.3389/fmed.2025.1731005

**Published:** 2026-01-12

**Authors:** Yuanyuan Zhao, Jun Shi, Hui Zhao, Letao Yu, Wei Wang, Changchang Hu, Liangliang Yin, Jiashuai Tian, Ju Li, Wei Wang

**Affiliations:** 1 Department of Anesthesiology, The First Hospital of Anhui University of Science and Technology, Huainan, China; 2 Department of Anesthesiology, The Affiliated Jiangning Hospital of Nanjing Medical University, Nanjing, China

**Keywords:** breastfeeding, cesarean section, hydromorphone, PCIA, postpartum depression

## Abstract

**Background:**

Postpartum depression (PPD) is a common mental health condition affecting 10–20% of women, particularly after cesarean delivery, which may hinder maternal recovery and breastfeeding. Hydromorphone, a semi-synthetic opioid with unique pharmacologic properties, has recently been applied in postoperative analgesia. This study aimed to evaluate the effects of hydromorphone-based patient-controlled intravenous analgesia (PCIA) on PPD and related maternal–infant outcomes after cesarean section.

**Methods:**

In this prospective, randomized, double-blind controlled trial, 120 parturients aged 20–40 years scheduled for elective cesarean section were randomly assigned to receive either sufentanil (Group S, *n* = 60, 2 μg/kg) or hydromorphone (Group H, *n* = 60, 0.2 mg/kg) for PCIA. A computer-generated block randomization sequence was used, and allocation concealment was ensured through a centralized randomization system. Both regimens included palonosetron (4 μg/kg) diluted with saline to 150 mL and identical pump settings. The primary outcome was the Edinburgh Postnatal Depression Scale (EPDS) score on postoperative day 42. Secondary outcomes included EPDS score on day 3, breastfeeding satisfaction at 72 h, Ramsay sedation scores at 24 and 48 h, resting and active Numeric Rating Scale (NRS) pain scores at 2, 6, 12, 24, and 48 h, rescue analgesia requirements, and adverse events within 48 h after surgery.

**Results:**

Compared with Group S, Group H had significantly lower EPDS scores on postoperative days 3 and 42, higher breastfeeding satisfaction at 72 h, and lower Ramsay sedation scores at 24 and 48 h (all *p* < 0.01). Resting and active NRS pain scores at 6 and 12 h were also reduced (*p* < 0.05). The incidence of dizziness and drowsiness within 48 h was significantly lower in Group H (*p* < 0.05). No significant differences were observed between the two groups in rescue analgesia requirements or other adverse events.

**Conclusion:**

Hydromorphone-based PCIA after cesarean section was associated with lower EPDS scores on postoperative days 3 and 42, improved breastfeeding satisfaction at 72 h, and a lower incidence of dizziness and drowsiness.

**Clinical trial registration:**

www.chictr.org.cn, identifier ChiCTR2500105264.

## 1 Introduction

Postpartum depression (PPD) is one of the most common psychological disorders during the perinatal period, with a global prevalence of approximately 17% (1). Several meta-analyses (2–4) have shown that cesarean section is associated with a higher risk of PPD compared with vaginal delivery (pooled OR 1.26–1.33). In addition to a history of depression or anxiety and inadequate social support, poor pain control during and after delivery, decreased sleep quality, and daytime dysfunction (e.g., somnolence or oversedation) have been independently associated with the development of PPD (5–7). PPD not only reduces the rate and duration of breastfeeding and weakens maternal–infant bonding but is also linked to impaired cognitive, emotional, and social development in infants (8). Therefore, evaluations of postoperative analgesic regimens after cesarean section should not be limited to pain intensity or opioid consumption but should also incorporate emotional outcomes as key endpoints, particularly within the current emphasis on multimodal and opioid-sparing perioperative analgesia (9).

Patient-controlled intravenous analgesia (PCIA) is widely used for postoperative pain management following cesarean delivery, despite increasing adoption of multimodal and opioid-sparing strategies, with opioids remaining a core component of PCIA in routine practice (10, 11). Sufentanil, a potent *μ*-opioid receptor agonist, provides effective analgesia but lacks evidence of mood-stabilizing or antidepressant effects. Conversely, its high protein-binding affinity and central accumulation can lead to adverse reactions such as dizziness and somnolence in some parturients (12, 13), resulting in fluctuating analgesic experiences and decreased alertness. These side effects may compromise maternal comfort and physiological stability, impeding emotional recovery and the establishment of early neonatal suckling behaviors (14, 15). Therefore, sufentanil-based regimens alone offer limited benefit for emotional outcomes, highlighting the need for analgesic strategies that better support mood regulation and breastfeeding outcomes.

Hydromorphone, a semi-synthetic opioid, acts as a potent *μ*-opioid receptor agonist and also shows moderate affinity for *δ*-opioid receptors (16). Previous studies have shown that hydromorphone has a relatively low protein-binding rate, a stable plasma free fraction, and few active metabolites, resulting in a rapid onset and sustained analgesic effect with good safety and efficacy (17). In addition, several reports suggest that hydromorphone may influence both pain perception and emotional modulation through *δ*-receptor pathways, which could help relieve postoperative depression, anxiety, and sleep disturbances observed in patients undergoing major surgery such as rectal cancer resection (18). However, evidence regarding the impact of hydromorphone-based patient-controlled intravenous analgesia (PCIA) on postpartum depression and breastfeeding outcomes after cesarean section is still scarce. Considering its pharmacological characteristics and findings from previous studies (17, 18), we hypothesized that hydromorphone-based PCIA could help reduce depressive symptoms in women following cesarean delivery. Therefore, we designed a prospective, randomized, double-blinded, controlled trial to compare hydromorphone- and sufentanil-based PCIA in terms of maternal mood and maternal–infant outcomes, with the aim of providing further evidence to guide postoperative analgesic strategies and reduce the risk of postpartum depression.

## 2 Methods

### 2.1 Participants

A total of 120 parturients scheduled for elective lower-segment cesarean section between July and September 2025 at the First Affiliated Hospital of Anhui University of Science and Technology (Huainan First People’s Hospital) were enrolled in this study. All participants had clear medical indications for cesarean delivery, including but not limited to uterine scarring, abnormal fetal position, cephalopelvic disproportion, or severe pregnancy-related complications.

The inclusion criteria were as follows: age between 20 and 40 years; singleton, full-term pregnancy; body mass index (BMI) between 19.1 and 39.5 kg/m^2^; and American Society of Anesthesiologists (ASA) physical status class II or III. The exclusion criteria were: (1) known allergy to opioids; (2) history of psychiatric disorders or current use of antidepressant medication; (3) history of substance or alcohol dependence; (4) preoperative Edinburgh Postnatal Depression Scale (EPDS) score ≥13 (19); (5) severe cardiac, pulmonary, hepatic, or renal dysfunction; (6) inability to complete the questionnaire assessments; (7) failure of neuraxial anesthesia requiring conversion to general anesthesia; (8) anesthetic block level (temperature sensory level) above T_4_ or too low to meet surgical requirements; (9) operation time >2 h or intraoperative blood loss >1,500 mL; and (10) occurrence of serious postoperative complications such as eclampsia or pulmonary embolism.

### 2.2 Sample size estimation

**Figure 1:**
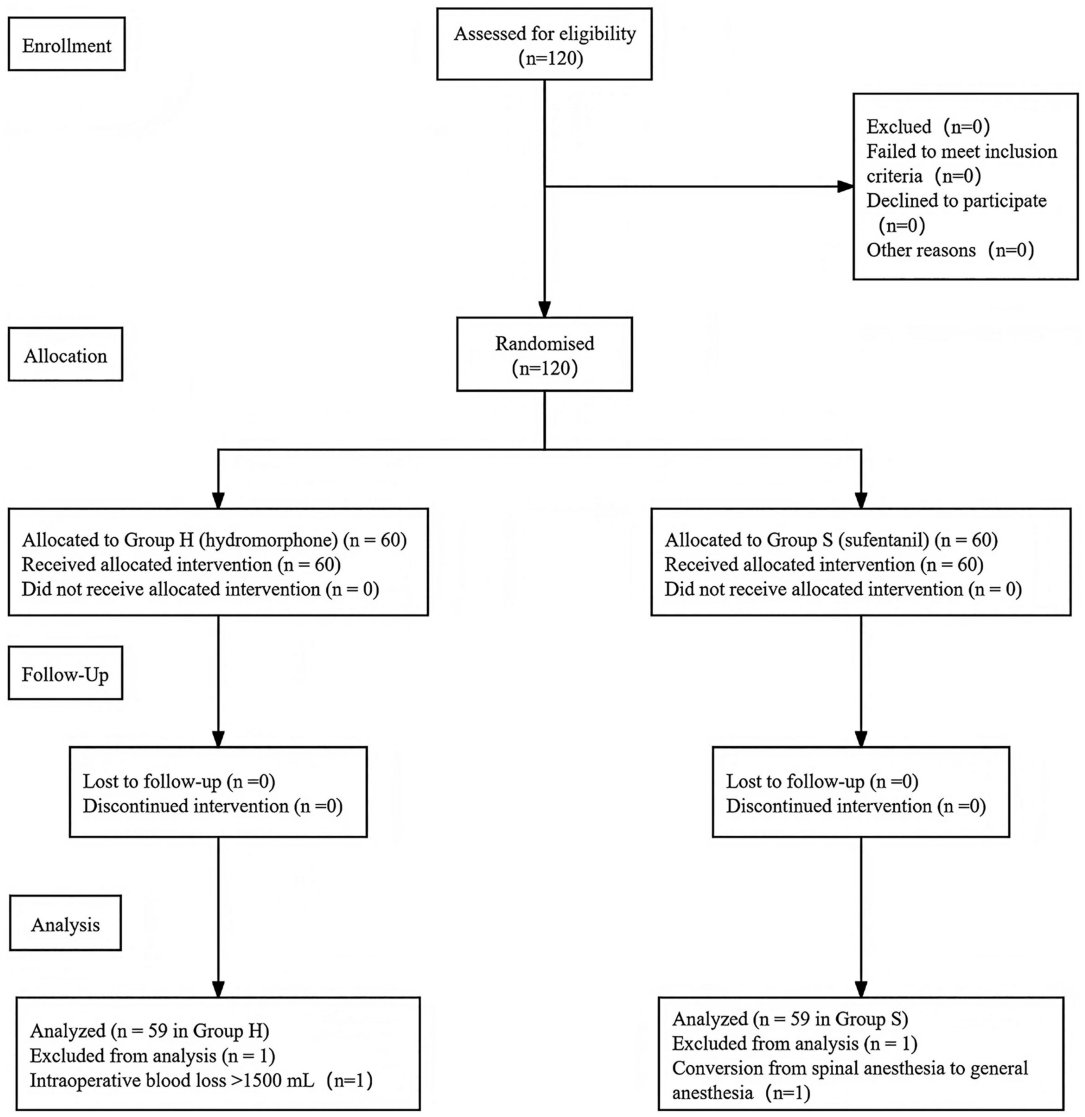
CONSORT flow diagram. CONSORT flow diagram illustrating participant enrollment, randomization, allocation, and analysis. All assessed participants met the eligibility criteria and were randomized. A total of 120 parturients were randomized; two were excluded (one due to conversion from spinal to general anesthesia, and one due to intraoperative blood loss >1,500 mL). Consequently, 118 participants (59 per group) were included in the per-protocol analysis.

The primary endpoint of this study was the Edinburgh Postnatal Depression Scale (EPDS) score on postoperative day 42. Based on data from previous studies (20, 21), the standard deviation (*σ*) of EPDS scores at this time point was approximately 4, and the expected difference in mean scores (*Δ*) between the two groups was 2. Assuming a two-sided *α* level of 0.05 and a *β* level of 0.20 (80% power), the required sample size was calculated to be 54 participants per group. Considering an anticipated dropout rate of 10%, the final total sample size was determined to be 120 participants, with 60 allocated to each group. The participant recruitment process is illustrated in Figure 1 (CONSORT flow diagram).

### 2.3 Randomization and allocation concealment

Participants were randomly allocated into two groups using a computer-generated block randomization sequence created with SPSS Statistics, version 26.0 (IBM Corp., Armonk, NY, USA). The randomization list was placed in sealed, opaque envelopes and kept by an independent research coordinator who had no role in the clinical procedures, ensuring proper allocation concealment. All anesthesia procedures were performed by the same anesthesiologist, and all cesarean sections were conducted by the same obstetric team. The patient-controlled analgesia (PCIA) pumps were prepared by one anesthesia nurse who was blinded to group allocation, with identical pump appearance and labeling. Both the patients and the outcome assessors were unaware of the treatment assignments. All study data were coded and analyzed anonymously, and blinding was maintained until the completion of the 42-day postoperative follow-up.

### 2.4 Interventions

At the end of surgery, all patients received an intravenous loading dose before the initiation of patient-controlled intravenous analgesia (PCIA). In the hydromorphone group (Group H), hydromorphone 5 μg/kg was diluted to 5 mL with normal saline and administered intravenously. In the sufentanil group (Group S), sufentanil 0.05 μg/kg was given in the same way. For postoperative analgesia, the PCIA solution in Group H contained hydromorphone 0.2 mg/kg and palonosetron 4 μg/kg, diluted with 0.9% saline to a total volume of 150 mL. In Group S, the mixture consisted of sufentanil 2 μg/kg and palonosetron 4 μg/kg, also diluted to 150 mL. The settings for the PCIA pumps were identical in both groups, with a background infusion rate of 2 mL/h, a bolus dose of 2 mL, a lockout interval of 15 min, and a total duration of 48 h.

### 2.5 Anesthesia management

All parturients fasted for 8 h and refrained from drinking for 4 h before surgery. After entering the operating room, an intravenous line was established, and standard monitoring was applied, including electrocardiography (ECG), heart rate (HR), noninvasive blood pressure (NIBP), and pulse oxygen saturation (SpO₂). Oxygen was delivered via nasal cannula at 2 L/min. Patients were positioned in the left lateral decubitus position, and combined spinal-epidural anesthesia (CSEA) was performed at the L_3_-L_4_ interspace. A dose of 12–15 mg of 1% ropivacaine was injected intrathecally, followed by placement of an epidural catheter advanced approximately 4 cm into the epidural space. Five minutes later, the sensory block level was assessed using a temperature test. Cesarean delivery was started when the block height reached between T_6_ and T_4_. If the block level was higher than T_4_ or remained inadequate despite additional epidural dosing, the patient was excluded from the study. During anesthesia, if the mean arterial pressure (MAP) decreased by more than 20% from baseline or the systolic blood pressure (SBP) fell below 90 mmHg, rapid fluid infusion was administered, and phenylephrine 50 μg was given intravenously to maintain hemodynamic stability. At the end of surgery, the patient-controlled intravenous analgesia (PCIA) pump was connected. Within 48 h after surgery, if the resting Numeric Rating Scale (NRS) score reached 4 or higher, intravenous flurbiprofen axetil 50 mg was provided as rescue analgesia, and each rescue event was recorded.

### 2.6 Outcomes

The primary outcome of this study was the Edinburgh Postnatal Depression Scale (EPDS) score on postoperative day 42.

Secondary outcomes included: (1) EPDS scores before surgery and on postoperative day 3; (2) breastfeeding satisfaction at 72 h postoperatively; (3) Ramsay sedation scores at 24 and 48 h after surgery; (4) resting and active Numeric Rating Scale (NRS) pain scores at 2, 6, 12, 24, and 48 h postoperatively; (5) the number of effective PCIA button presses and cases requiring rescue analgesia within 48 h after surgery; and (6) the incidence of adverse events within 48 h, including dizziness, drowsiness, postoperative nausea and vomiting (PONV), respiratory depression (defined as SpO₂ < 90% lasting for ≥10 s or respiratory rate < 8 breaths/min) (22), and pruritus.

#### 2.6.1 EPDS assessment

Postpartum depressive symptoms were assessed using the EPDS scale, a 10-item self-report screening tool designed to evaluate the severity of depressive symptoms in the postpartum period. Each item is rated on a 4-point scale (0–3), yielding a total score ranging from 0 to 30, with higher scores indicating greater depressive symptom burden. In accordance with prior literature, scores of 0–9 indicate minimal depressive symptoms, 10–12 suggest an increased screening-defined risk, and scores ≥13 indicate a high risk warranting further clinical evaluation (19).

#### 2.6.2 Breastfeeding satisfaction

Breastfeeding satisfaction was assessed 72 h postoperatively using a locally developed 5-point Likert scale based on five dimensions—milk volume, ease of breastfeeding, infant cooperation, maternal comfort, and satisfaction with nursing support. Each item was scored from 1 to 5, with a total score range of 5–25. Higher scores indicated greater breastfeeding satisfaction (23). This scale was used for exploratory and descriptive assessment of early breastfeeding experience in the immediate postoperative period.

#### 2.6.3 Ramsay sedation scale

A score of 1 indicates anxiety, agitation, or restlessness; 2 denotes alertness and cooperation; 3 reflects response only to verbal commands; 4 corresponds to response to light glabellar tap or loud auditory stimulus; 5 represents no response to strong stimuli (deep sleep); and 6 indicates no response and unarousable deep sedation. Scores below 2 suggest inadequate sedation, scores between 2 and 4 indicate appropriate sedation, and scores of 5–6 denote oversedation or somnolence with a potential risk of respiratory depression (24).

Dizziness and drowsiness were assessed based on patient self-report and routine clinical assessment, without the use of additional standardized diagnostic criteria.

### 2.7 Statistical analysis

Statistical analyses were performed using SPSS version 26.0 (IBM Corp., Armonk, NY, USA). The Shapiro–Wilk test was applied to assess the normality of continuous variables. Normally distributed data were expressed as mean ± standard deviation (SD) and compared between groups using the independent samples t-test. Non-normally distributed data were presented as median (interquartile range, IQR) and analyzed using the Mann–Whitney U test. Categorical variables were expressed as numbers and percentages [*n* (%)] and compared using the chi-square test or Fisher’s exact test, as appropriate. A two-sided *p* value < 0.05 was considered statistically significant. The final analysis was conducted on a per-protocol basis, excluding participants who met predefined exclusion criteria after randomization.

## 3 Results

### 3.1 Enrollment of participants

A total of 120 parturients were randomized after eligibility assessment, with no participants excluded prior to randomization. After randomization, two participants were excluded according to the predefined criteria: one in the sufentanil group due to conversion from spinal anesthesia to general anesthesia, and another in the hydromorphone group because of intraoperative blood loss exceeding 1,500 mL. Consequently, 118 participants completed the study and were included in the final analysis, with 59 in the sufentanil group and 59 in the hydromorphone group (Figure 1).

### 3.2 Baseline characteristics

Patients in both groups had similar demographic and perioperative characteristics, including maternal age, gestational age, BMI, ASA physical status classification, duration of surgery, intraoperative blood loss, intraoperative fluid volume, and neonatal Apgar scores at 5 min after birth (all *p* > 0.05, Table 1).

**Table 1:** Patient demographic and perioperative data.

Variable	Group S (*n* = 59)	Group H (*n* = 59)	*p* value
Age, year (mean ± SD)	31.1 ± 5.0	30.9 ± 5.1	0.830
BMI, kg/m^2^ (mean ± SD)	28.6 ± 3.7	28.4 ± 4.0	0.761
Gestational age, day (mean ± SD)	273.2 ± 9.0	270.9 ± 7.6	0.180
Class of ASA, *n* (%)			0.648
I	57 (96.6)	56 (94.9)	
II	2 (3.4)	3 (5.1)	
Duration of surgery, min (mean ± SD)	46.9 ± 10.2	47.3 ± 9.1	0.788
Intraoperative blood loss, mL [*M* (IQR)]	300 (200, 300)	300 (200, 300)	0.581
Intraoperative fluid infusion, mL [*M* (IQR)]	650 (650, 700)	650 (650, 750)	0.207
Neonatal Apgar score [*M* (IQR)]	9 (9, 10)	9 (9, 10)	0.195

Data are shown as mean ± SD, *M* (IQR), or *n* (%). A *p*-value < 0.05 was considered to be statistically significant. SD, standard deviation; BMI, body mass index; ASA, American Society of Anesthesiologists.

### 3.3 Comparison of EPDS, Ramsay scores, and breastfeeding satisfaction between groups

Compared with Group S, the Group H showed statistically lower EPDS scores on postoperative days 3 and 42 (*p* < 0.01) and lower Ramsay sedation scores at 24 and 48 h postoperatively (*p* < 0.01). Furthermore, breastfeeding satisfaction scores at 72 h after surgery were significantly higher in Group H than in Group S (*p* < 0.01) (Table 2).

**Table 2:** EPDS, Ramsay scores and breastfeeding satisfaction.

Variables		Group S (*n* = 59)	Group H (*n* = 59)	*p* value
EPDS score	Preoperative	8.3 ± 1.4	8.4 ± 1.3	0.342
	Postoperative day 3	7.9 ± 1.0	6.6 ± 1.0	<0.001
	Postoperative day 42	6.2 ± 0.9	5.4 ± 1.0	<0.001
Ramsay sedation score	24 h after surgery	2.4 ± 0.5	2.1 ± 0.4	0.001
	48 h after surgery	2.2 ± 0.4	2.0 ± 0.2	0.001
Breastfeeding satisfaction score	72 h after surgery	18.1 ± 1.3	19.0 ± 1.5	0.001

Data are shown as mean ± SD. A *p*-value < 0.05 was considered to be statistically significant. EPDS, Edinburgh Postnatal Depression Scale; SD, standard deviation.

### 3.4 Postoperative pain

Compared with Group S, Group H showed significantly lower NRS pain scores at rest and during movement at 6 and 12 h postoperatively (*p* < 0.05). There were no significant differences between the two groups in NRS pain scores at 2, 24, and 48 h, the number of rescue analgesia cases, or the effective PCIA button presses within 48 h after surgery (Table 3).

**Table 3:** Postoperative NRS scores and analgesic consumption.

Variables	Time point	Group S (*n* = 59)	Group H (*n* = 59)	*p* value
Resting NRS score [points, *M* (IQR)]	2 h after surgery	2.0 (1.0, 2.0)	2.0 (1.0, 2.0)	0.389
	6 h after surgery	2.0 (2.0, 3.0)	2.0 (1.0, 3.0)	0.013
	12 h after surgery	2.0 (2.0, 3.0)	2.0 (1.0, 2.0)	0.032
	24 h after surgery	1.0 (1.0, 2.0)	1.0 (1.0, 2.0)	0.137
	48 h after surgery	1.0 (1.0, 2.0)	1.0 (1.0, 1.0)	0.172
Dynamic NRS score [points, *M* (IQR)]	2 h after surgery	2.0 (1.0, 2.0)	2.0 (1.0, 2.0)	0.336
	6 h after surgery	3.0 (3.0, 4.0)	3.0 (2.0, 3.0)	0.024
	12 h after surgery	3.0 (3.0, 3.0)	3.0 (2.0, 3.0)	0.022
	24 h after surgery	2.0 (1.0, 2.0)	2.0 (1.0, 2.0)	0.225
	48 h after surgery	1.0 (1.0, 2.0)	1.0 (1.0, 2.0)	0.239
Rescue analgesia [*n* (%)]		5 (9.0)	6 (10.0)	0.751
Effective PCA attempts within 48 h (mean ± SD)		3.7 ± 1.2	4.2 ± 0.7	0.706

Data are shown as *M* (IQR) or mean ± SD, as appropriate. A *p*-value < 0.05 was considered to be statistically significant.

NRS, numerical rating scale; PCA, patient-controlled analgesia; SD, standard deviation.

### 3.5 Adverse events

Compared with Group S, Group H showed significantly lower incidences of dizziness and drowsiness within 48 h after surgery (*p* < 0.05). There were no significant differences between the two groups in the incidence of postoperative nausea and vomiting (PONV), respiratory depression, or pruritus within 48 h postoperatively (Table 4).

**Table 4:** Postoperative adverse events.

Variables	Group S (*n* = 59)	Group H (*n* = 59)	*p* value
Dizziness [*n* (%)]	10 (16.9)	2 (3.4)	0.033
Drowsiness [*n* (%)]	6 (10.2)	0	0.027
PONV [*n* (%)]	5 (8.5)	4 (6.8)	0.729
Respiratory depression [*n* (%)]	0	0	
Pruritus [*n* (%)]	2 (3.4)	0	0.497

Data are shown as *n* (%). A *p*-value < 0.05 was considered to be statistically significant. PONV, postoperative nausea and vomiting.

## 4 Discussion

In recent years, increasing attention has been given to the relationship between postoperative pain management and psychological well-being in women undergoing cesarean section. The choice of analgesic regimen after delivery not only affects the quality of pain control but may also influence emotional status, sleep quality, and breastfeeding outcomes (25, 26). The results of this study showed that, compared with sufentanil-based PCIA, hydromorphone-based PCIA was associated with statistically lower EPDS scores on postoperative days 3 and 42, higher breastfeeding satisfaction, and a lower incidence of dizziness and drowsiness, while providing comparable analgesic efficacy.

Currently, the main modalities of postoperative analgesia after cesarean section include patient-controlled epidural analgesia (PCEA) and patient-controlled intravenous analgesia (PCIA) (27). Compared with PCEA, PCIA has been more widely used because of its advantages such as ease of operation, rapid onset of analgesia, avoidance of neuraxial puncture, and elimination of catheter-related complications. Commonly used agents for PCIA include opioid analgesics such as fentanyl and sufentanil. Several studies (28, 29) have demonstrated that these agents effectively relieve postoperative pain and improve maternal comfort after cesarean section without affecting breastfeeding. Sufentanil is a highly selective *μ*-opioid receptor agonist with substantially greater analgesic potency than both morphine and hydromorphone. Pharmacodynamic studies have shown that its analgesic efficacy is approximately 500–1,000 times that of morphine and 50–100 times that of hydromorphone, with a wide therapeutic window and predictable pharmacokinetic properties (30). Previous studies reported that sufentanil at doses of 2–3 μg/kg provides effective postoperative analgesia for cesarean delivery with a relatively low incidence of adverse reactions (31). Hydromorphone is a semi-synthetic opioid receptor agonist widely used for postoperative PCIA, with doses of approximately 0.1–0.2 mg/kg providing effective analgesia and acceptable safety (32). Based on these data, we selected 2 μg/kg sufentanil and 0.2 mg/kg hydromorphone to compare analgesic and emotion-related outcomes during PCIA after cesarean section. Comparable analgesic efficacy was observed across postoperative time points, supporting the clinical comparability of the two regimens with respect to pain control. However, as equianalgesic equivalence between different opioids is difficult to define, potential pharmacodynamic differences, including sedation profiles, should be considered when interpreting emotion-related outcomes.

PPD is one of the major factors affecting maternal and infant health. Previous studies have reported that approximately 10–20% of women develop depressive symptoms to varying degrees during the early puerperium (33). Without timely intervention, PPD can seriously disrupt the mother–infant bond, impair breastfeeding, and hinder neonatal development (34). Evidence suggests that postoperative day 3 represents a peak period for the onset of “baby blues” and serves as a critical window for predicting PPD. An Edinburgh Postnatal Depression Scale (EPDS) score of ≥9 is considered an early warning sign, with a sensitivity of up to 88% (35–37). Furthermore, the period between 4 and 6 weeks postpartum is recognized as a key window for the clinical diagnosis of PPD (38). Therefore, postoperative days 3 and 42 were selected as the observation time points for EPDS assessment in the present study, aligning with both the temporal characteristics of the disorder and clinical relevance.

Our findings showed that EPDS scores in the hydromorphone group were statistically lower than those in the sufentanil group at both time points. However, the absolute magnitude of this difference was modest, and mean EPDS scores in both groups remained below commonly used screening thresholds, indicating a low-risk study population. Prior research has shown that combining esketamine with sufentanil for postoperative PCIA after cesarean section reduces the incidence of PPD at 1 and 6 weeks in a dose-dependent manner, whereas sufentanil alone has limited effect (39). Similarly, a multicenter study demonstrated that administering 0.2 mg/kg esketamine immediately after delivery reduced the incidence of major depressive episodes at 42 days from 25.4 to 6.7%, with mean EPDS scores decreasing by approximately 3 points at both 7 and 42 days postpartum (40). Compared with these findings, the present results suggest that hydromorphone-based PCIA may be associated with a mild reduction in postpartum depressive symptom burden, rather than a definitive prevention of postpartum depression, highlighting that different opioid-based analgesic strategies may be associated with distinct emotional outcomes during the early postpartum period.

A prospective randomized study in non-obstetric patients reported that hydromorphone-based intravenous analgesia improved mood states at 48 and 96 h after rectal cancer surgery compared with sufentanil (18). Although the evaluation tools and time points differed, these results support the potential mood-stabilizing properties of hydromorphone. Pharmacologically, hydromorphone is a semi-synthetic opioid agonist that acts primarily on *μ*-opioid receptors but also exhibits partial affinity for *δ* receptors, which are involved in emotional regulation and mood-related effects (41). Previous experimental and clinical studies have shown that δ-receptor agonists play a significant role in modulating mood, alleviating anxiety, and exerting antidepressant-like effects (42, 43). Moreover, hydromorphone maintains stable plasma free concentrations and produces fewer active metabolites, minimizing central accumulation and opioid-related adverse reactions (44, 45), which may indirectly contribute to improved maternal comfort and emotional stability.

In recent years, the concept of “psychoceuticals” has gained attention in perioperative medicine, referring to pharmacological agents that may exert short-term modulatory effects on mood and emotional recovery. In obstetric anesthesia, however, the clinical use of such strategies remains limited and is generally considered adjunctive (46). Low-dose ketamine or esketamine has been explored in this context for its potential to attenuate acute stress responses and depressive symptoms following cesarean delivery. Although these approaches were not incorporated into the present study, our findings suggest that optimization of opioid-based PCIA may serve as a practical adjunct to routine perioperative care aimed at supporting early postpartum emotional recovery.

In addition to pharmacological approaches, block-based analgesic strategies have been increasingly explored as opioid-sparing options in perioperative pain management. In routine cesarean practice, however, the feasibility and consistency of these techniques vary across institutions and depend on local expertise and resources. Recent studies have shown that regional anesthesia techniques may reduce postoperative opioid requirements and improve analgesic quality, with potential downstream effects on stress-related and emotional outcomes (47). In cesarean delivery, interfascial plane blocks and related regional techniques have been investigated as adjuncts to multimodal analgesia, aiming to improve maternal comfort during the early postoperative period. Although such block-based strategies were not included in the present protocol, they may represent a useful complementary option alongside optimized PCIA in future clinical studies.

In addition to emotional outcomes, this study also evaluated breastfeeding performance. Physiologically, the onset of lactogenesis II typically occurs within 24–72 h after delivery, and delayed initiation has been associated with lower rates of exclusive breastfeeding and shorter breastfeeding duration (48). Accordingly, breastfeeding satisfaction was assessed at 72 h postoperatively, as this time point coincides with the typical establishment of lactogenesis II and represents a critical window for early breastfeeding initiation and maternal adaptation. Women who undergo cesarean section have been shown to experience a lower incidence of lactogenesis II initiation within the first 72 h postpartum compared with those who deliver vaginally (49). Karlström et al. (50) reported in a cohort study that greater postoperative pain negatively affects mother-infant contact, latching, and breastfeeding posture, suggesting that improved analgesia and maternal comfort may be associated with better early breastfeeding experience and satisfaction. Similarly, a double-blind randomized controlled trial involving 160 women undergoing elective cesarean delivery demonstrated that optimizing the sedation-analgesia experience was associated with early breastfeeding-related indicators (51).

In the present study, mothers in the hydromorphone group reported higher breastfeeding satisfaction at 72 h postoperatively. This finding may partly reflect reduced pain-related interference with breastfeeding posture and latching, as well as differences in early emotional well-being. Breastfeeding satisfaction is influenced by multiple psychosocial and neonatal factors, including social support, marital status, education level, sleep quality, and neonatal conditions, the present results suggest that postoperative analgesic strategies may play a meaningful role in shaping early breastfeeding experience.

A meta-analysis including 13 studies with a total of 812 patients reported that the median plasma protein binding rate of hydromorphone was significantly lower than that of sufentanil (11.6% vs. 88.4%), and that the free drug concentration of hydromorphone remained stable throughout the course of patient-controlled analgesia (17). In contrast, the unbound fraction of sufentanil gradually increased with the duration of treatment. This pharmacokinetic difference may explain why hydromorphone exhibits stronger analgesic efficacy during the early postoperative phase, whereas its advantage diminishes within 24–48 h as the free sufentanil concentration rises.

Consistent with these findings, our study demonstrated that the hydromorphone group had significantly lower NRS pain scores at 6 and 12 h postoperatively compared with the sufentanil group, indicating a faster onset and more effective early analgesia (32, 52). No significant differences were observed at 2, 24, or 48 h after surgery, which may be attributed to residual spinal anesthesia at 2 h and the use of equipotent doses between the two groups, potentially masking the difference in analgesic effects (53).

Manabe et al. (54) reported that hydromorphone exhibits a distinct G-protein–biased agonism, preferentially activating G-protein signaling pathways associated with analgesia while minimally engaging *β*-arrestin-mediated pathways. This pharmacological feature may contribute to reduced respiratory depression and other adverse events, distinguishing it from conventional opioids and providing a mechanistic basis for its favorable safety profile.

Additionally, previous studies have shown that the protein binding of sufentanil is markedly influenced by plasma concentration and fluid balance, leading to fluctuations in the unbound fraction, whereas hydromorphone maintains a relatively constant protein-binding rate and stable pharmacokinetics (55). These properties may allow hydromorphone to provide more consistent and sustained analgesia, particularly in patients with complex or chronic pain conditions.

Regarding safety, our results demonstrated that the incidence of dizziness and drowsiness as well as Ramsay sedation scores within 48 h postoperatively, were lower in the hydromorphone group than in the sufentanil group. This may be related to its lower protein binding and steadier plasma concentration (18). No significant differences were observed in nausea, vomiting, respiratory depression, pruritus, or the need for rescue analgesia between groups, further supporting the clinical feasibility and safety of hydromorphone in PCIA after cesarean delivery.

Postpartum depression arises from a complex interplay of behavioral, psychosocial, and biological factors. In addition to pharmacological and analgesic strategies, early supportive measures and acute-phase indicators—such as sleep quality, anxiety-related symptoms, or stress responses—may help characterize emotional vulnerability during the early postpartum period; however, these aspects were not assessed in the present study.

This study has several limitations. Firstly, this was a single-center study with a relatively small sample size conducted in a specific clinical setting, which may limit the generalizability of the findings, particularly to higher-risk populations or emergency cesarean deliveries. Secondly, although the EPDS is a widely used screening tool, it does not provide a psychiatric diagnosis or biological correlates and primarily reflects depressive symptom burden or risk. While statistically significant differences in EPDS scores were observed between groups, the magnitude of change did not reach commonly reported thresholds for minimal clinically important difference, which may reflect the low-risk nature of the study population with low baseline EPDS scores, the indirect and modest emotional effects of a postoperative analgesic intervention, and the limited sample size. Thirdly, breastfeeding satisfaction was assessed using a locally developed Likert-type scale designed to capture early postoperative breastfeeding experience; however, formal psychometric validation of this instrument has not yet been performed, which may affect the comparability of breastfeeding-related findings across studies.

Future multicenter studies incorporating psychosocial variables and multidisciplinary assessments are warranted to further clarify the external validity and clinical implications of these findings.

## 5 Conclusion

In conclusion, compared with sufentanil, hydromorphone-based PCIA provided comparable postoperative analgesia and was associated with lower EPDS scores, higher breastfeeding satisfaction, and a lower incidence of dizziness and drowsiness following cesarean section. Given the modest absolute differences in EPDS scores, these findings indicate an association with reduced depressive symptom burden rather than a definitive reduction in clinically diagnosed postpartum depression.

## Data Availability

The raw data supporting the conclusions of this article will be made available by the authors, without undue reservation.
